# Erroneous Communication Messages on COVID-19 in Africa

**DOI:** 10.4269/ajtmh.20-0540

**Published:** 2020-06-03

**Authors:** Bernard Seytre

**Affiliations:** bnscommunication, Paris, France

## Abstract

Adherence of the population to COVID-19 prevention recommendations is crucial to control the epidemic. However, a study of communication messages around COVID-19 in 15 West African countries showed a number of unfounded messages, as well as a lack of communication on critical information to understand the prevention measures being promoted. Incidents of violence that have taken place recently suggest that general mistrust and hostility could grow, similar to the events that occurred during the previous Ebola epidemics. It is therefore urgent to review and revise the COVID-19 communication messages currently used in sub-Saharan Africa.

Control of the COVID-19 epidemic is especially important in sub-Saharan Africa, where most countries have under-resourced health systems operating at maximum capacity, with a large existing workload in hospitals and clinics.^1^ Population compliance with prevention measures against COVID-19 is an important part of disease control, and this compliance requires that people understand the prevention measures and trust the political and health authorities promoting them. A study I recently conducted as part of an assignment for the West African Health Organization, an Economic Community of West African States (ECOWAS) institution, showed that, on the one hand, some of the information being communicated on COVID-19 is scientifically unfounded, whereas on the other, crucial information to explain transmission of the virus and the way to avoid it are missing.

Doubt and hostility among the population profoundly hampered efforts to control the recent Ebola epidemics in the Democratic Republic of Congo and West Africa. Doubts around COVID-19 prevention are currently widespread in Africa, and acts of hostility have already occurred. These have included the destruction of a COVID-19 testing center suspected of disseminating the disease in Abidjan; the attack of a health center and police station by fishermen in Sierra Leone after a limitation of the number of boats allowed to go out to fish; and a taxi-driver strike over restrictions on taxi use in Guinea, which was followed a few weeks later by demonstrations against travel restrictions, during which several protesters were killed by the police. The potential for growth of doubt, mistrust, and hostility should be a major concern for the control of the COVID-19 epidemic.

I reviewed the content of messages on COVID-19 transmitted by various communication tools and aimed at the general population, which I was able to download from official Health Ministry websites and Facebook pages for the 15 countries belonging to ECOWAS. This review included 148 posters and/or flyers and 38 videos and/or audio spots, with an average of 12.4 documents per country, ranging from two for Sierra Leone to 33 for Benin (Table 1). There were two limitations to this review. First, it was possible that certain communication messages created by the Health Ministries were not available online. Second, it was not possible to determine which communication documents are actually being used. However, the sampling was large enough to be representative of the communication messages used in these countries.

**Table 1:** Documents downloaded from websites or Facebook pages of the Ministries of Health

	Posters and flyers	Videos* and radio spots
Benin	31	2
Burkina Faso	7	4
Cabo Verde	24	4
The Gambia	1	2
Ghana	5	1
Guinea	12	2
Guinea Bissau	6	0
Ivory Coast	13	2
Liberia	4	0
Mali	18	8
Niger	1	1
Nigeria	15	4
Senegal	6	4
Sierra Leone	1	1
Togo	4	3
Total	148	38

* The same video in different languages is counted once only, as are similar videos with different traditional leaders.

This analysis showed that very few communication messages focused on the virus responsible for the outbreak. In countries where health literacy is low, how can people understand that common objects such as door handles can transmit the disease if they are not told that the virus (or microbe) that causes this disease can be deposited onto objects? How can they understand that speaking with someone can transmit the disease? Health authorities should not expect that people will just trust and follow their recommendations. Recommendations that are not fully explained or understood are likely to raise doubts and suspicions.

Of the 15 countries surveyed, 11 had communication messages warning against an alleged risk for COVID-19 from contact with wild and domestic animals (mammals, fish, birds, and reptiles). For example, “*It* [COVID-19] *has been found in many animals including bat, cat, camel and cattle*” (Liberia); “*Thorough cooking of meat and eggs*” and “*No unprotected contact with live wild or farm animals*” (Nigeria); “*Thoroughly cook meat, fish and eggs before eating them*” (Mali); “*Avoid unprotected contact with wild or breed animals and with surfaces in contact with them*” (Togo); “*When visiting fairs and markets, avoid direct contact with live animals and surfaces where live animals have been handled*” (Guinea Bissau). These messages were illustrated with drawings of animals and/or food (bats, chicken, pigs, ham, steaks, fish, eggs, pangolins, grasscutters, or snakes). Among the documents I examined, those from only four countries (Benin, the Gambia, Ghana, and Sierra Leone) did not have these messages.

The WHO Regional Office for Africa also provides communication tools carrying messages suggesting risks of COVID-19 from animal contact. Among the six posters on the “*Coronavirus (COVID-19)/Protect yourself*” page of its website, one, entitled “*How to protect yourself*,” lists six recommendations of which one advises: “*Avoid raw meat/live animals*” (Figure 1). The same page features two videos with “*tips for protecting against the coronavirus*” of which one tip advises: “*Avoid direct contact with live animals. If this is impossible, make sure to clean your hands afterwards”* (illustrated with a chicken and a pig); and another states: “*Do not eat raw or poorly cooked animal products and wash your hands, clean surfaces and utensils afterwards*”

**Figure 1.**
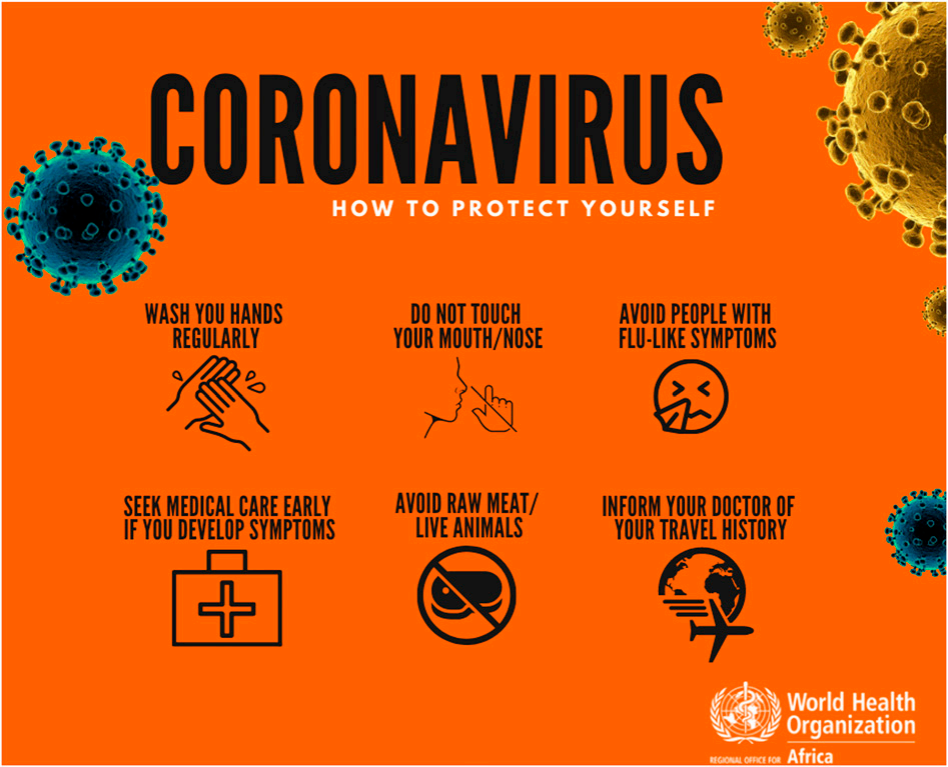
One of the six “social media cards” available on the WHO Regional Office for Africa website.

These messages have no scientific basis, as the animal spillover of the coronavirus that causes COVID-19 was presumably a unique event that occurred in China, probably from a bat species that is not present in Africa.^2^ Not only are these messages misleading, but they can potentially raise hostilities.

An anthropological study we conducted in 2015 in Togo on the representations of Ebola and the perception of information communicated showed that the most well-known messages were those banning game hunting and consumption.^3^ These messages were unfounded, as the spillover of the Ebola virus from bats had been a unique event, which likely occurred once for each Ebola epidemic. In countries such as Togo, Ivory Coast, and Liberia, where these messages were promoted, people continued hunting without contracting Ebola, leading to questions raised as to why hunting was prohibited.^4^ Our study showed that these messages stirred up various theories and suspicions and contributed to mistrust of the health authorities.

In contrast to the West Africa Ebola epidemic, no country has prohibited hunting during the present COVID-19 outbreak, yet the current messages warn about contact with all animal species, including not only wild animals but also domestic animals, fish, and eggs. People with access to the Internet are able to access information on the verified COVID-19 means of transmission and to see that no country outside sub-Saharan Africa carries these warnings. Furthermore, farmers will keep taking care of their cattle and people will keep eating meat, fish, and eggs as usual without getting sick. This is a major cause for distrust, which could extend to the accurate messages about necessary prevention measures.

Finally, although many of the communication tools I reviewed describe the symptoms of COVID-19, only one message, a video from the Malian Ministry of Health, explained that individuals can transmit the disease without being symptomatic, which is the very reason for the general confinement—otherwise isolating only those who are sick would be sufficient. Without this crucial information, how can people understand confinement? Once again, not fully understanding the rationale behind the rules imposed by the authorities, often under the threat of fines or jail, is likely to raise mistrust.

Accurate communication messages may not be enough to eliminate the numerous rumors in circulation about COVID-19 in ECOWAS and elsewhere or to ensure full compliance with the prevention measures in place among populations. However, at the very least, these messages should avoid fueling mistrust and doubt in the community. It is urgent to review and revise current communication around COVID-19 to remove erroneous messages and provide accurate and necessary information. Finally, it would be worth studying whether the erroneous messages in circulation originated from the WHO Regional Office for Africa and whether they are being used in other African countries.
